# The Mediating Role of Social Camouflaging on the Relationship Between Autistic Traits and Orthorexic Symptoms

**DOI:** 10.3390/brainsci15050503

**Published:** 2025-05-14

**Authors:** Barbara Carpita, Benedetta Nardi, Cristiana Pronestì, Gianluca Cerofolini, Matilde Filidei, Chiara Bonelli, Gabriele Massimetti, Ivan Mirko Cremone, Stefano Pini, Liliana Dell’Osso

**Affiliations:** Department of Clinical and Experimental Medicine, University of Pisa, 56126 Pisa, Italy; barbara.carpita@unipi.it (B.C.); cristianapronesti@gmail.com (C.P.); gianlucacerofolini@gmail.com (G.C.); m.filidei@studenti.unipi.it (M.F.); chiarabonelli.95@hotmail.it (C.B.); gabriele.massimetti@unipi.it (G.M.); ivan.cremone@gmail.com (I.M.C.); stefano.pini@unipi.it (S.P.); liliana.dellosso@unipi.it (L.D.)

**Keywords:** camouflaging, autistic traits, orthorexia, orthorexia nervosa, eating disorder

## Abstract

**Background/Objectives**: Recent lifestyle and dietary changes, driven by health awareness and ecological concerns, have led to the rise in numerous type of diets, which can promote well-being but may also contribute to Orthorexia Nervosa (ON), which have been suggested to be linked to autism spectrum disorder. This study aimed to explore the relationship between autistic traits, social camouflaging, and orthorexic tendencies in female university students, focusing on how these factors intersect with specific dietary habits. **Methods**: 554 female students were recruited via an online survey and assessed with the Adult Autism Subthreshold (AdAS) Spectrum, the Camouflaging Autistic Traits Questionnaire (CAT-Q), and the ORTO-R. Participants were categorized into four groups based on AdAS Spectrum and CAT-Q quartiles. **Results**: Vegans and vegetarians exhibited higher orthorexic tendencies and specific autistic traits. High scorers on the AdAS Spectrum and CAT-Q also showed higher ORTO-R scores, with both AdAS Spectrum and CAT-Q total scores, as well as certain domains, serving as significant positive predictors of higher ORTO-R scores. Notably, the AdAS Spectrum total score had a significant direct and indirect effect (through the CAT-Q) on the ORTO-R total score. **Conclusions**: The study found significant associations between autistic traits, social camouflaging behaviors, and orthorexic tendencies in female university students. These findings suggest that the strict dietary behaviors and rigid thinking characteristic of orthorexia may be influenced by underlying autistic features, highlighting the need for further research into the intersection of autism and eating disorders.

## 1. Introduction

In recent years, lifestyle and dietary habits have shifted significantly, particularly in Western populations [1,2]. Advances in medicine, improved socioeconomic conditions, and the rise in (social) media have contributed to a growing focus on health and well-being. This includes increasing attention to fitness, balanced nutrition, and the avoidance of foods high in preservatives, calories, and unhealthy fats [3,4]. Concurrently, environmental, ethical, and sustainability concerns have further driven changes in dietary practices [5,6,7]. As a result, many individuals have adopted dietary patterns such as Mediterranean, vegetarian, and vegan diets, with their prevalence influenced by geographic and cultural contexts [8,9]. While these trends are often beneficial—being associated with reduced risk for cardiovascular disease and cancer [10,11,12] and promoted by institutions like the WHO [13]—they may also carry unintended psychological consequences. One such concern is Orthorexia Nervosa (ON), a term coined by Bratman in 1997 to describe an unhealthy obsession with eating only “pure” or “correct” foods [14]. Since its introduction, numerous studies have explored ON, but its diagnostic criteria remain contested, and it is not yet officially recognized in the major international mental health classification systems. Despite its growing visibility, ON remains a controversial construct, lacking official diagnostic recognition and suffering from measurement inconsistencies. Available data suggest that the prevalence of ON is rising, although it varies significantly, ranging from 2.6% to as high as 87.7% [15,16]. The considerable variability in ON prevalence rates is largely attributed to inconsistencies in its measurement, particularly with the widely used ORTO-15. While the ORTO-15 helped spark early interest in orthorexic behaviors, it has been heavily criticized for its poor psychometric properties and lack of diagnostic precision. Crucially, it struggles to distinguish between health-conscious eating and pathological obsession, often inflating its prevalence by capturing non-clinical behaviors. Moreover, its reliability and factor structure vary significantly across languages and cultural contexts, further undermining its validity. The absence of standardized diagnostic criteria for ON compounds these issues, making it difficult to determine which symptoms warrant clinical concern. In contrast, newer tools like the Dusseldorf Orthorexic Scale (DOS) and the Bratman Orthorexia Self-Test (BOS-T) show improved construct validity and cross-cultural consistency, highlighting the need to move beyond outdated instruments and toward more reliable measures of orthorexic pathology.

Certain groups, including vegetarians, vegans, individuals following specific diets, athletes, cancer survivors, postpartum women, and females, are at greater risk [15,16]. The ongoing research indicates that orthorexia exists on a spectrum within the general population, extending from individuals who engage in healthy eating behaviors to those who follow highly restrictive diets with potentially harmful medical consequences [16,17]. For this reason, some researchers distinguish between “healthy” and “unhealthy” orthorexia, while others prefer to stage the ON according to a multidimensional profile, considering varying degrees of orthorexic tendencies [14,18,19]. Individuals with ON typically prioritize the quality of food over its taste, focusing intensely on all aspects of food, including its preparation, the ritual of meals, and the selection of raw ingredients [14]. This mindset often leads to rigid and inflexible eating habits that can disrupt their entire lifestyle. Such restrictive behaviors frequently interfere with social interactions, as individuals may avoid dining out at restaurants or eating away from home [14,20]. Furthermore, individuals with strong orthorexic tendencies often view their self-imposed, supposedly healthier eating patterns as morally superior, which can create a divide between themselves and others, fostering a dichotomous mindset [21,22].

The primary clinical features of ON, including restrictive dietary habits, an obsessive focus on food, and ritualized eating behaviors, have led researchers to debate whether ON should be classified as a Feeding and Eating Disorder (FED) or whether it represents a form of Obsessive–Compulsive Disorder (OCD) [23,24,25]. Some studies suggest that ON is more likely to be classified within the FEDs spectrum, while others have observed shared tendencies toward obsessionality between ON, FEDs, and OCD [21,26]. The connection between ON and FEDs is supported by findings that individuals with FEDs, particularly those with anorexia nervosa (AN), exhibit more orthorexic tendencies than the general population [27,28,29]. Indeed, ON and AN share various clinical features and personality traits, including a higher prevalence among females, restrictive dietary behaviors, ritualized eating patterns, cognitive rigidity, perfectionism, a drive for thinness, and body dissatisfaction [30,31,32]. However, ON and AN differ in terms of concerns related to body weight and shape, as well as the typically lower Body Mass Index (BMI) in AN patients, reflecting the differing motivations behind food restriction—health and ethical reasons in ON versus weight loss in AN [33]. Nevertheless, these differences may be explained by the influences of the cultural context on personal beliefs, and by the evolution of clinical manifestations of AN throughout history [21]. According to this hypothesis, in a society that identifies healthy and ethical living as a personal added value, while continuing to stigmatize body image-centered eating disorders [34,35], ON could represent an emerging, mostly accepted, form of FEDs, specifically a new expression of AN.

Nowadays, a growing body of research is focusing on the investigation of autistic features—defined autistic traits (ATs)—in clinical populations, and particular interest has been given to the assessment of ATs in FEDs, in particular AN [36,37]. Interestingly, several studies have identified a link between ON and autism spectrum disorder (ASD), paralleling the well-established association between ASD and AN [38]. ASD is a neurodevelopmental condition characterized by persistent deficits in social communication and interaction, restricted and repetitive interests and behaviors [39]. Clinical manifestations of ASD vary largely along the “spectrum”, spacing from severe intellectual and language impairment to “high-functioning” form, where individuals possess good social networks and can maintain self-sufficiency in work and daily life [39]. It is now recognized how ASD and autistic features exist on a continuum within the general population, from typically neurodeveloped individuals to those with mild or isolated symptoms and to those exhibiting more pronounced features of the disorder [40]. While the current literature suggests that the prevalence of ASD is under 1%, it is likely underestimated due to the subtlety of some clinical manifestations, which may not emerge until social demands surpass an individual’s capabilities [39,41]. However, to date, numerous studies have demonstrated the importance of evaluating ATs even when they do not fully meet the diagnostic criteria for they represent a heightened risk of developing psychiatric disorders, experiencing traumatic events, and facing suicidality, especially after encountering emotional or physical stressors [42,43]. Furthermore, individuals with high-functioning ASD or ATs often employ strategies to mask their social deficits, known as “social camouflaging”. Although camouflaging occurs in both sexes, it is supposed to be more commonly and effectively used by females [44,45,46]. Indeed, females with ASD tend to exhibit milder social impairments and greater coping abilities, resulting in fewer diagnoses compared to their male counterparts (with a male-to-female ratio of 3:1) [47].

Given these overlaps, several researchers have explored the link between ASD and AN, with some proposing AN as a potential female-specific phenotype of autism [37,48,49]. Traits such as rumination, cognitive inflexibility, social anhedonia, and impaired theory of mind are shared by both conditions [49,50,51,52]. In this framework, although the association between ON and ATs has been explored less, some researchers have also considered the possibility that ON, a relatively new form of FED closely related to AN, may reflect an underlying autistic vulnerability [21]. Indeed, cognitive rigidity and sensory sensitivities, two hallmark features of ASD, may underlie the inflexible eating behaviors and heightened attention to food quality often seen in ON. Supporting this notion, a few earlier studies—despite being underpowered—have noted a psychopathological overlap between autism spectrum and ON, as well as an increased prevalence of ATs in individuals exhibiting high orthorexic tendencies, with female sex and high ATs being predictive of ON [22,38,53,54].

Building on this framework, the present study aimed to examine the presence of ATs, social camouflaging, and orthorexic tendencies among a female university population, focusing on exploring possible intertwined relationships among these dimensions. Additionally, the study evaluated the association between the investigated psychopathological dimensions and a specific dietary habit.

## 2. Materials and Methods

### 2.1. Study Sample and Procedures

For the purpose of the study, a sample of 554 female students, under the age of 23 and enrolled in three-year degree coursers, was evaluated in the framework of a project featuring an invitation from the Atheneum by email. Students who agreed to participate anonymously fulfilled through an online form the Adult Autism Subthreshold Spectrum (AdAS Spectrum), the Camouflaging Autistic Traits Questionnaire (CAT-Q), and the ORTO-R. The recruitment and assessment processes were authorized by the Ethics Committee of the Azienda Ospedaliero-Universitaria of Pisa, and the study was carried out in compliance with the Declaration of Helsinki.

### 2.2. Measures

#### 2.2.1. The AdAS Spectrum

The AdAS Spectrum is a 160-item self-report instrument used to evaluate a broad range of autism-related symptoms in people who do not have cognitive or linguistic deficits. The questionnaire is divided into seven domains investigating different areas: Childhood and Adolescence, Verbal Communication, Non-verbal Communication, Empathy, Inflexibility and adherence to routine, Restricted interests and Rumination and Hyper- and Hyporeactivity to Sensory Input. During the validation study, the questionnaire revealed strong internal consistency, exceptional test–retest reliability, and convergent validity with other dimensional measures of autism [55].

#### 2.2.2. The CAT-Q

The CAT-Q is a questionnaire, recently validated in its Italian version, designed to measure social camouflaging behaviors characteristic of subjects with autism spectrum disorder. The questionnaire comprises twenty-five items arranged in three domains—Assimilation, Masking, and Compensation—rated on a seven-point Likert scale. During the validation study, the instrument showed excellent internal consistency, with the Italian version showing a Cronbach’s alpha of 0.904, as well as test–retest reliability and convergent validity with additional ASD measures [56,57].

#### 2.2.3. The ORTO-R

The ORTO-R [58] is a revised, shortened version of the ORTO-15 [59], a questionnaire designed to assess ON symptoms. It contains 6 items, each rated on a Likert scale. Unlike the ORTO-15, where lower scores indicated more severe ON symptoms, the ORTO-R uses higher scores to indicate a stronger tendency toward ON. The ORTO-R was developed by reevaluating the ORTO-15’s original validation data, addressing its reliability issues and aligning it with updated ON definitions. Validation studies have shown that the ORTO-R is a reliable tool with good psychometric properties.

The ORTO-R offers an improvement over the ORTO-15 by overcoming the latter’s limitations, and it is considered a more accurate measure of ON symptoms. This makes the ORTO-R a valuable tool in research, offering a more up-to-date and reliable alternative to older versions of the questionnaire. Following the procedure reported in previous studies [60], participants fulfilled the ORTO-15 and for the aims of this work we analyzed only the items included in the ORTO-R and revised the scores in order to report them as ORTO-R score.

### 2.3. Statistical Analysis

Initially, student *t*-tests were employed to compare scores across the AdAS Spectrum, CAT-Q, and ORTO-R domains, based on the participants’ dietary regimes. The sample was then divided into two groups according to AdAS Spectrum quartiles: one group included participants who scored above the third quartile (“AdAS high scorers”, subjects in fourth quartile reported an AdAS Spectrum total score > 67), while the other comprised those who scored below it (“AdAS low scorers”, subjects in fourth quartile reported a CAT-Q total score > 100). Similarly, participants were categorized into CAT-Q quartiles, resulting in “CAT-Q high scorers” for those above the third quartile and “CAT-Q low scorers” for those below. A *t*-test was performed in order to compare ORTO-R scores between AdAS spectrum high and low scorers and CAT-Q high and low scorers.

To further investigate the effect of being above the third quartile of the AdAS Spectrum and CAT-Q scores on ORTO-R scores, a two-way factorial analysis of variance (ANOVA) was conducted. Subsequently, a linear regression analysis was performed, treating the AdAS Spectrum and CAT-Q total scores as independent variables and the ORTO-R total score as the dependent variable. This analysis aimed to determine whether the total scores of the questionnaires could statistically predict higher ORTO-R scores. Given that both independent variables showed a significant association with the ORTO-R total score, a mediation analysis was carried out using the AdAS Spectrum total score as the predictor, the ORTO-R total score as the dependent variable, and the CAT-Q total score as the mediator. The Hayes PROCESS tool was utilized, and bootstrap confidence intervals for both unstandardized and standardized indirect effects were computed.

Two additional linear regression analyses were then conducted. The first analyzed the ORTO-R total score as the dependent variable, with CAT-Q domain scores as independent variables, to assess whether these domain scores could predict higher ORTO-R scores. The second analysis used the same dependent variable but included AdAS Spectrum domain scores as independent variables to evaluate their predictive capability for higher ORTO-R scores.

All statistical analyses were performed with SPSS version 26.0.

## 3. Results

The sample recruited consisted of 554 female university students with a mean age of 20.54 ± 1.41, enrolled in three-year university programs at three prestigious Italian universities. The majority of participants, 498 (89.9%), reported following an omnivorous diet, while 30 (5.4%) identified as vegetarian, and 8 (1.4%) as vegan. Additionally, 18 participants (3.2%) chose not to disclose their dietary habits.

Results from the student *t*-test indicated that participants adhering to vegetarian or vegan diets scored significantly higher on the AdAS Spectrum Restricted interests and Ruminations domain as well as on the overall ORTO-R score, compared to their omnivorous counterparts (see Table 1).

Moreover, both AdAS Spectrum and CAT-Q high scorers showed higher ORTO-R scores than low scorers (see Table 2).

The ANOVA analysis revealed a significant main effect of belonging to the group that scored above the third AdAS Spectrum quartile on the ORTO-R total score, and an almost significant effect of belonging to the CAT-Q high scorers. However, the interaction between these two variables did not yield a significant effect on the ORTO-R score (see Table 3).

The linear regression was conducted using ORTO-R total score as dependent variable and AdAS Spectrum and CAT-Q total scores as independent variables. Results showed that both the AdAS Spectrum and CAT-Q total scores were significant positive predictors of a higher ORTO-R total score (see Table 4).

**Figure 1:**
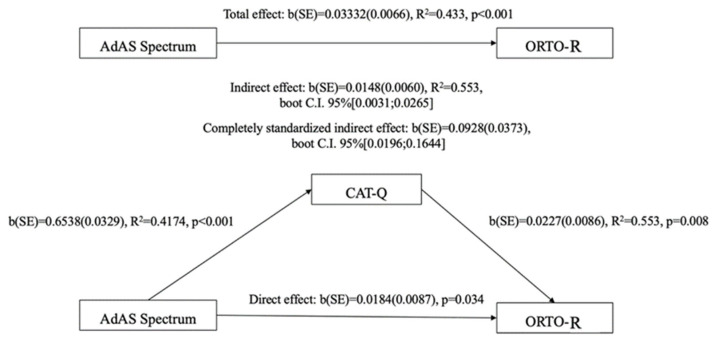
Mediation analysis results.

The results from the Mediation Analysis demonstrated significant total and direct effects of the AdAS Spectrum total score on the ORTO-R total score, with a total effect of 0.0333 (*p* < 0.001) and a direct effect of 0.018 (*p* = 0.034). Furthermore, the AdAS Spectrum total score also exhibited a significant indirect effect on the ORTO-R total score through the CAT-Q total score (b = 0.015, 95% bootstrapped CI [0.003:0.026]), meaning that ATs also influence orthorexic tendencies indirectly by increasing camouflaging behavior (CAT-Q), which in turn is related to more orthorexic behaviors. The standardized indirect effect, or index of mediation, was b = 0.093, with a 95% bootstrapped CI [0.020:0.164] (see Figure 1).

Subsequently, we conducted two additional linear regression analyses. The first analysis, which used the ORTO-R total score as the dependent variable and CAT-Q domain scores as independent variables, revealed that the CAT-Q domains of Compensation and Masking were statistically significant positive predictors of the ORTO-R total score (see Table 5). In the second analysis, which also treated the ORTO-R total score as the dependent variable but utilized AdAS domain scores as independent variables, the results indicated that the Non-Verbal Communication domain was a positive predictor of the ORTO-R total score, while the Verbal Communication domain acted as a negative predictor (see Table 6).

## 4. Discussion

The aim of the present work was to evaluate the relationship between autism spectrum, including social camouflaging behaviors, and orthorexic tendencies in a sample of female university students. We also evaluated eventual correlates of specific dietary habits.

Our results highlighted how the prevalent dietary regime was omnivorous. Moreover, meat-avoiders (individuals who followed a vegetarian or vegan diet) scored significantly higher on the ORTO-R questionnaire compared to omnivorous participants. This finding is overall in line with the available literature, as some previous investigations found a correlation between ON or orthorexic tendencies, and a peculiar diet styling, including vegetarianism and veganism [61,62,63]. Indeed, meat-avoidance dietary behaviors share some common features with ON, including food restrictions, leading some authors to consider being vegetarian or vegan as a risk factor for the development of ON, as well as other FEDs [64,65].

However, to date, there is currently no clear scientific evidence to support the notion that vegetarians and vegans are more likely than omnivores to engage in FEDs [66,67]. For instance, an important limitation of the majority of studies that examines the association between FED symptoms and meat avoidance, is that they tend to lump all meat-avoiders together, without distinguishing between different dietary regimes [68,69].

On the other hand, results associating a meat-avoidant dietary regime and orthorexic tendencies appear to be more consistent. According to preliminary data, vegans and vegetarians appear to be more likely than omnivores to report being health-conscious [70], and with many of them claiming that health and health-related issues played a role in their decision to follow the diet [71]. Upon this evidence, some authors suggested that those who follow dietary regimes that cut out substantial categories of macronutrients may be more likely to develop ON because their obsession with food may become excessive [72]. These hypotheses and our results are supported by recent studies that reported how vegans and vegetarians appear to be at higher risk ON than meat-eaters [73,74,75,76].

In particular, an extensive review from Mathieu et al. that systematically analyzed the studies published from 2000 to 2022 found convergent results on lower BMI levels and higher rates of ON in vegetarian/vegan samples, as compared to the omnivores [9]. Nevertheless, there is a source of confusion concerning affirmations based on the arbitrary threshold and cutoffs pre-settled in the variety of scales used to measure orthorexic features. For instance, Heiss and co-workers demonstrated how the ORTO-R score could overestimate ON among a specific subgroup of subjects like vegans [77].

Notably, our results also highlighted that vegetarian or vegan individuals scored significantly higher in the AdAS Spectrum Restricted interests and Rumination domain compared to those following an omnivorous diet. Interestingly, although some studies have investigated the presence of cognitive inflexibility and perseverative thinking among subjects with different dietary regimes, including vegetarians/vegans, to date, the available data do not allow us to draw definitive conclusions [78,79]. However, the clinical overlap between these specific dietary habits and tendency towards ruminative thinking could be explained by the fact that most of the subjects strictly adhering to a considered “healthy” and “sustainable” diet, have been reported to often show a perseverative focus on food, and its correlates [70].

Furthermore, frequently, mainly in vegans, this specific interest can involve several different areas of life, assuming the form of a real lifestyle [70]. In this framework, our results may also reflect overlapping dimensions of ethical rigidity and ATs. These individuals may adhere to restrictive diets not only for health but also for moral or ideological reasons, consistent with rigid, rule-governed behavior often observed in individuals with high ATs. Speculatively, the intense focus on ethical or health-related dietary choices could serve as a socially acceptable outlet for cognitive inflexibility or rumination, thereby linking ON with autism-related thinking styles. Thus, the dietary and lifestyle inflexibility expressed in ON and vegetarians/vegans could reflect an intrinsic tendency to repetitive thinking and behaviors in these subjects, to the point of leading some authors to hypothesize a significant correlation between ON and Obsessive–Compulsive disorder [80]. On the other hand, the same inflexibility and repetitive thinking, and the presence of a strict focus on food, health or ethical concerns, rituals in food intake, and perfectionism, observed in subjects with ON has also been related to the presence of concomitant ATs [38].

Moreover, according to our results, subjects with higher AdAS scores also reported greater ON symptoms, and a significant effect of the AdAS Spectrum score on ORTO-15 total score was highlighted. In addition, results from the linear regression analysis reported the AdAS Spectrum total score as a significant positive predictor of higher ORTO-R scores. These findings are in line with those of a previous study that reported the same effect of ATs on the scoring of ORTO-R, particularly in females [22]. A direct or indirect correlation between autistic features and orthorexic tendencies has been variously reported in the available literature [22,38,54,55], and could be explained by several factors.

Firstly, subjects with ON may show ritualized behaviors linked to food intake, which could be mimicking the autistic strict adherence to routine and repetitive behavioral patterns. Similarly, rigid thinking regarding health concerns, expressed in ON, may reflect the cognitive inflexibility characterizing subjects on the spectrum, while persistent focus on food and lifestyle could be a manifestation of the presence of specific and restricted interests, as well as repetitive thinking in ON subjects.

Moreover, ON individuals often present strict moral principles and feelings of moral superiority, which can lead to manifestations of intolerance toward different eating attitudes considered “incorrect”, and this aspect may be related to a social–emotional reciprocity impairment, also typical of the autistic spectrum [21,22].

Moreover, the link between ON and autistic features is also sustained by the evidence that patients suffering from ASD frequently show restricted and selective dietary patterns, on a different basis in different subjects [21,81]. Furthermore, there is an extensive overlapping symptomatology between ON and other FEDs, including AN [38,80], and a large number of studies is reporting a close relationship between AN and the autism spectrum to the point that some authors proposed that AN could be reconceptualized as a female autism spectrum phenotype [48,50,82,83,84].

Thus, while several commonalities between AN and ON as different variants of restrictive eating disorders have been highlighted, the presence of significant ATs could represent a common psychopathological dimension underlying both ON and AN, manifested through restricted interests on food and dietary patterns, especially in female patients [21,22,38,85].

Interestingly, also subjects with higher CAT-Q scores reported higher ON symptoms, and the CAT-Q total score also showed a quasi-significant effect (*p* = 0.52) on ORTO-R scores, when applying the ANOVA analysis. Interestingly, this lack of significance for the interaction between AdAS Spectrum and CAT-Q scores, suggests that the effects of the two variables on orthorexic tendencies are additive rather than synergistic. In addition, the linear regression revealed a positive predictive factor of CAT-Q on ORTO-R scores. Moreover, the result of the mediation analysis, showed that besides a direct effect of the AdAS Spectrum on the total ORTO-R score, there is also an indirect effect of autism spectrum on ON mediated by the CAT-Q, indicating that the adoption of camouflaging strategies, eventually aimed at emulating the interest and behaviors of neurotypical peers, as well as masking and shaping their core autistic symptoms in order to make them more socially acceptable, may partially act as a mediating factor in the relationship between ATs and orthorexic tendencies. This result may additionally support the connection between autistic features and the development of orthorexic tendencies, as CAT-Q measures the presence of camouflaging behaviors proper of the autistic spectrum. Camouflaging strategies are usually employed by subjects on the autistic spectrum, especially women, as a coping strategy to compensate for their self-perceived social difficulties [43,57]. Through camouflaging behaviors, individuals with high levels of ATs or ASD can mask their communicative and social impairments, and the greater use of camouflaging among females may be at least partially responsible of the underdiagnosis of ASD in this gender [86,87]. While, noticeably, both social camouflaging and FEDs have been linked to female phenotypes of autism spectrum, our results are in line with a previous study that highlighted how social camouflaging may predict eating disorder symptoms in autistic adults. [84,87,88,89]. The basis of the link between social camouflaging and eating disorder symptoms is not clear and should be further investigated. It could be hypothesized that subjects with high levels of ATs, while adopting camouflaging strategies to adjust themself to the environmental contest, may assume a highly rigid and selective dietary behaviors, also under the influence of cultural inputs and social media campaigns [22,55,89]. Platforms such as Instagram, TikTok, and YouTube have become powerful drivers of health and wellness trends, frequently idealizing restrictive dietary practices under the guise of “clean eating” [90]. These trends often promote binary thinking around food (e.g., clean vs. dirty, pure vs. toxic), which may reinforce rigid behaviors in vulnerable individuals, including those with high levels of cognitive inflexibility or a tendency toward obsessive thinking. In this context, social media could act as a competing or complementary pathway to ON, either independently or in interaction with neurodevelopmental traits like those seen in ASD [91,92,93,94,95]. For example, individuals who engage in camouflaging to fit social norms may be especially drawn to emulate idealized eating behaviors widely shared and validated online [96]. Through stressing these positive objectives in their lifestyle, these subjects could attribute meanings of “pure/impure” or “healthy/unhealthy” to the food, alongside developing pathological/orthorexic behaviors which are, paradoxically, dangerous for their own health [22,38,55].

However, it should be noted that the effect size of the mediation analysis is relatively small. Such a modest effect may indicate that camouflaging behaviors play only a limited role in explaining the link between ATs and ON. Nevertheless, even small effects can be clinically relevant when considered in the context of multifactorial psychological phenomena, particularly those involving complex interactions between personality traits, social adaptation strategies, and environmental influences. Thus, although the effect size is limited, it may still hold conceptual and preventative importance—particularly for identifying at-risk individuals before symptoms intensify.

It is also worth noting that the effect of the CAT-Q total score on ORTO-R scores in the ANOVA analysis approached but did not reach conventional significance (*p* = 0.052). While the mediation model as a whole was statistically supported through bootstrapped indirect effects, this marginal *p*-value underscores the need for caution in interpreting the strength of the mediation. It is possible that the indirect effect of camouflaging is subtle and may only manifest more clearly in larger or more clinically diverse samples. Alternatively, this result may reflect variability in how camouflaging operates across individuals—with some using it adaptively, and others experiencing it as a source of psychological burden that contributes to disordered eating. Future research should explore these individual differences to better delineate the precise role camouflaging plays in the ON–autism spectrum relationship.

In support of these results, linear regression analyses highlighted CAT-Q Compensation and Masking domains and AdAS Spectrum Non-verbal Communication domain as significant positive predictors of ON tendencies. Interestingly, these results are consistent with each other. Indeed, CAT-Q Compensation and Masking domains explore two camouflaging strategies aimed at reducing others’ perception of one’s neuroatypicality. In particular the Compensation domain specifically examines ways to actively compensate for social challenges, such as mimicking and practicing others’ body language or facial expressions, whereas the Masking domain looks into ways to conceal Ats, such as keeping an eye on and modifying your body and face to seem at ease or engaged [57]. On the other hand, the AdAS Spectrum Non-verbal Communication domain investigates the subject’s perception of his/her own difficulties in social and non-verbal communication, which may also drive the individuals to implement camouflaging strategies such as compensation and masking, in order to mask their neuroatypicality [97,98,99]. Therefore, women with high levels of autistic features in domains involving social deficiencies, could be better able to identify their social challenges and eventually hide them by implementing camouflage strategies, which, in this case, could focus on imitative behaviors regarding socially accepted (and in some cases even valorized) topics such as diet [84,100,101,102,103]. In this perspective, ON may be viewed as a manifestation of the same autism spectrum phenotype. The underlying autistic features may constitute the symptomatologic core of the disorder, showing up as a particular emphasis on food and nutrition [21,83]. The severity and type of clinical picture may be influenced by the specific inputs received by the social environment, which may shape the differences in the subjective mentalization of symptoms, for example, embracing restrictive dietary practices based on personal convictions regarding the “healthiness” of foods [21,83].

Moreover, our results highlighted AdAS Spectrum Verbal Communication domain as a negative predictor of ON tendencies. Interestingly, this result is concordant with those of a previous study carried in a non-clinical sample, where deficits in verbal communications negatively predicted the presence of orthorexic symptoms [38]. This result may be linked to the fact that, as previously exposed, in the female sex, ATs usually manifest with a more preserved social functioning. In this framework, when a more severe impairment of verbal communication is present, it is more likely to not be associated with the particular autism phenotype that underlies dietary restriction (such as ON), which instead may show with a more preserved social and communication functioning, with increased awareness of non-verbal communication deficit but more conserved language abilities [104].

From a clinical perspective, our findings suggest that the assessment of ATs, particularly camouflaging behaviors, may be a valuable component of early detection in individuals presenting with orthorexic tendencies. Given that both ATs and camouflaging were positively associated with ON symptoms, clinicians should consider systematically screening for these features—especially in female patients, who may be more likely to mask neurodevelopmental differences through socially adaptive behaviors such as dietary restriction. Camouflaging behaviors, in particular, may obscure the presence of underlying ATs, delaying accurate diagnosis and potentially leading to misattribution of symptoms to other psychiatric conditions. Early identification of these neurodevelopmental features could facilitate more individualized treatment approaches that address not only eating behaviors but also the cognitive and social coping mechanisms contributing to their maintenance.

### Limits

These results should be seen in light of some limitations. For instance, the cross-sectional design of the study prevents us from making temporal or causal connections between the variables under analysis. Moreover, the self-report nature of the psychometric questionnaires used for the evaluation of the participants may induce an over- or under-estimation of symptoms based on the subjective perception. In addition, the recruitment procedure, which included exclusively University students, limits the extensibility of our results to other populations. This lack of diversity in the sample limits generalizability to other demographics such as males, older adults, or non-student populations. Also, biases related to sample selection may be considered, such as an overrepresentation of subjects who were more interested in the topic. Moreover, since participation was voluntary, respondents may have been more health-conscious or psychologically aware, which ultimately may have skewed our results. Lastly, although the ORTO-R represents an improvement over the original ORTO-15, it remains a relatively new tool and retains some of the limitations of its predecessor. Notably, it has been criticized for its ongoing difficulty in distinguishing between genuinely health-conscious eating and pathological preoccupation with healthy food, potentially inflating prevalence rates by capturing non-clinical behaviors, especially in certain populations with specific dietary habits such as vegans and vegetarians. Additionally, its reliability and factor structure have shown considerable variability across different languages and cultural contexts, raising concerns about its overall validity.

## 5. Conclusions

Our study identified significant associations between ATs, social camouflaging behaviors, and orthorexic tendencies among female university students, offering novel insights into the intersection of neurodevelopmental features and disordered eating patterns. Our findings suggest that traits commonly associated with autism may contribute to the development or maintenance of orthorexic behaviors, particularly in populations where such traits are often under-recognized or masked. Importantly, the role of social camouflaging emerged as a relevant mediator, implying that individuals who consciously or unconsciously mask their autistic characteristics to fit into social norms may be more vulnerable to internalizing behaviors like orthorexia. These results underscore the importance of adopting a neurodiversity-informed perspective when assessing and treating disordered eating, especially in females who may present with subtle or atypical manifestations of autism.

## Figures and Tables

**Table 1:** Comparison of scores obtained in the AdAS Spectrum, CAT-Q and ORTO-R questionnaires based on dietary habits.

	Omnivorous	Vegetarian/Vegan	t	*p*	Cohen’s d
**AdAS Spectrum**					
**Childhood adolescence**	7.22 ± 3.92	7.79 ± 4.10	−1.143	0.254	−0.192
**Verbal Communication**	5.51 ± 3.13	6.21 ± 3.12	−1.131	0.184	−0.224
**Nonverbal Communication**	10.31 ± 4.95	11.63 ± 5.96	−1.562	0.119	−0.263
**Empathy**	2.94 ±2.08	2.66 ± 2.35	0.802	0.423	−0.135
**Inflexibility and adherence to routine**	13.24 ± 6.91	15.08 ± 8.00	−1.564	00.118	−0.263
**Restricted interests and rumination**	8.13 ± 4.27	10.18 ± 4.63	−2.838	0.005 *	−0.478
**Hyper/Hypo-reactivity to sensory input**	4.33 ± 3.28	5.24 ± 3.41	−1.627	0.104	−0.274
**AdAS Spectrum total score**	51.69 ± 23.08	58.97 ± 25.25	−1.863	0.063	−0.314
**CAT-Q**					
**Compensation**	24.75 ± 9.52	23.79 ± 9.85	0.601	0.548	0.101
**Masking**	31.26 ± 7.86	31.63 ± 8.67	−0.280	0.780	−0.047
**Assimilation**	26.84 ± 10.46	27.68 ± 10.15	−0.482	0.630	0.081
**CAT-Q total score**	82.85 ± 23.65	83.10 ± 25.43	0.063	0.949	−0.011
**ORTO-R**					
**ORTO-R total score**	13.57 ± 3.78	14.63 ± 3.31	−1.680	0.001 *	−0.283

* Significant for *p* < 0.05.

**Table 2:** Comparison of ORTO-R scores between AdAS spectrum and CAT-Q high and low scorers.

	**AdAS High Scorers** **(N = 132)**	**AdAS Low Scorers** **(N = 422)**	**t**	***p***	**Cohen’s d**
ORTO-R	14.73 ± 3.89	13.28 ± 3.64	−3.93	<0.001 *	−0.392
	**CAT-Q High Scorers** **(N = 135)**	**CAT-Q Low Scorers** **(N = 419)**	**t**	***p***	**Cohen’s d**
ORTO-R	14.70 ± 3.64	13.28 ± 3.72	−3.88	<0.001 *	−0.384

* Significant for *p* < 0.05.

**Table 3:** Two-way ANOVA analysis with high/low scoring on AdAS Spectrum and CAT-Q as independent variables and ORTO-R total score as a dependent variable.

Source	Type III Sum of Squares	df	Mean Square	F	*p*
**Corrected Model**	308.426	3	102.809	7.594	<0.001 *
**Intercept**	61,119.312	1	61,119.312	4514.753	<0.001 *
**AdAS Spectrum 3 quartile**	60.201	1	60.201	4.447	0.035 *
**CAT-Q 3 quartile**	51.184	1	51.184	3.781	0.052
AdAS Spectrum 3 quartile * CAT-Q 3 quartile	26.930	1	26.930	1.989	0.159
**Error**	7445.728	550	13.538		
**Total**	110,565.000	554			
**Corrected total**	7754.153	553			

R^2^ = 0.040; adjusted R^2^ = 0.035; * Significant for *p* < 0.05.

**Table 4:** Linear regression analysis with ORTO-R total score as a dependent variable and AdAS Spectrum and CAT-Q total scores as independent variables.

	b (SE)	BETA	t	*p*	C.I. 95%	
					Lower Bound	Upper Bound
***constant***	**10.777 (0.563)**		**19.126**	<0.001 *	9.670	11.883
**AdAS Spectrum total score**	0.018 (0.009)	0.115	2.127	0.034 *	0.001	0.035
**CAT-Q total score**	0.023 (0.009)	0.144	2.647	0.008 *	0.006	0.039

R^2^ = 0.055; adjusted R^2^ = 0.052; Durbin-Watson: 1.52; * Significant for *p* < 0.05.

**Table 5:** Linear regressions analysis with ORTO-R total score as a dependent variable and CAT-Q domains as independent variables.

	b (SE)	BETA	t	*p*	C.I. 95%	
					Lower Bound	Upper Bound
***constant***	**10.335 (0.636)**		**16.253**	<0.001 *	9.086	11.585
**Compensation**	0.050 (0.023)	0.127	2.213	0.027 *	0.006	0.094
**Masking**	0.071 (0.026)	0.150	2.771	0.006 *	0.021	0.121
**Assimilation**	−0.006 (0.019)	−0.017	−0.313	0.755	−0.044	0.032

R^2^ = 0.057; adjusted R^2^ = 0.052; Durbin-Watson: 1.50; * Significant for *p* < 0.05.

**Table 6:** Linear regressions analysis with ORTO-R total score as a dependent variable and AdAS Spectrum domains as independent variables.

	b (SE)	BETA	t	*p*	C.I. 95%	
					Lower Bound	Upper Bound
***constant***	**11.786 (0.389)**		**30.280**	<0.001 *	11.021	12.551
**Childhood adolescence**	−0.002 (0.051)	−0.002	−0.037	0.971	−0.103	0.099
**Verbal Communication**	−0.209 (0.071)	−0.176	−2.929	0.004 *	−0.349	−0.069
**Nonverbal Communication**	0.163 (0.050)	0.219	3.267	0.001 *	0.065	0.262
**Empathy**	−0.131 (0.087)	−0.074	−1.510	0.132	−0.301	0.039
**Inflexibility and adherence to routine**	0.069 (0.036)	0.131	1.934	0.054	−0.001	0.140
**Restricted interests and rumination**	0.106 (0.060)	0.123	1.768	0.078	−0.012	0.224
**Hyper/Hypo-reactivity to sensory input**	−0.026 (0.066)	−0.023	−0.399	0.690	−0.155	0.103

R^2^ = 0.081; adjusted R^2^ = 0.069; Durbin-Watson: 1.55; * Significant for *p* < 0.05.

## Data Availability

All data generated or analyzed during this study are included in this published article.
